# Ureteric Leiomyosarcoma

**DOI:** 10.7759/cureus.49758

**Published:** 2023-11-30

**Authors:** Anupama Bahadur, Rajlaxmi Mundhra, Anoosha K Ravi, Poonam Gill, Anjali Pathak, Gayatri Suresh, Shreya Singhvi, Bhawana Mallick, Ashok Singh, Shalinee Rao

**Affiliations:** 1 Obstetrics and Gynecology, All India Institute of Medical Sciences, Rishikesh, Rishikesh, IND; 2 Pathology, All India Institute of Medical Sciences, Rishikesh, Rishikesh, IND

**Keywords:** spindle cell tumor, adnexal mass, sarcoma, ureter, leiomyosarcoma

## Abstract

Ureteric leiomyosarcoma is a rare but aggressive tumor among other sarcomas. There is no established epidemiological data due to the scarcity of literature on this uncommon disorder. The present literature comprises about 20 case reports mostly of women above 40 years of age. The presenting complaint is mostly pain in the abdomen with only a few reporting urological symptoms like hematuria. Understandably, this tumor is diagnosed by histopathological examination with immunohistochemistry. We report one such case of a 32-year-old female who underwent an exploratory laparotomy with preoperative suspicion of adnexal neoplastic mass only to find normal ovaries and left ureteric tumor intraoperatively. She was managed with excision of the tumor with partial resection of the involved ureter and end-to-end anastomosis of the ureter followed by chemotherapy and radiation.

## Introduction

Leiomyosarcoma is a form of soft tissue sarcoma, which makes room for a list of rare disorders. According to the Surveillance, Epidemiology, and End Results (SEER) data from 2017, nearly one out of 1,00,000 people are diagnosed with leiomyosarcoma every year. In general, leiomyosarcoma can occur at any site, mostly the uterus, stomach, and retroperitoneum, and is common in the elderly age group. Ureteric leiomyosarcoma is rare with just 20 cases reported to date [1,2]. The etiology, diagnostic modalities, and treatment are still a topic of debate as the available literature is scarce. Diagnosis and treatment of ureteric leiomyosarcoma in women need a multidisciplinary approach with gynecologists, radiologists, urologists, pathologists, and oncologists. Here, we report a 32-year-old female who presented with suspicion of an adnexal mass turning into an astonishing histopathological diagnosis of ureteric leiomyosarcoma after surgery.

## Case presentation

A 32-year-old Para 2 Live 2 was referred to our institute with complaints of pain in the abdomen for the past 10 days. She gave a history of laparotomy with left cystectomy for the left tubo-ovarian mass done two months ago in another hospital, but no histopathology report was available. She did not have bowel, bladder, or menstrual complaints. She exhibited a good performance status. On examination, an infraumbilical vertical scar was observed with an 18-20-week sized firm to hard abdominopelvic mass palpable in the suprapubic region with restricted mobility. Per vaginally, the uterus was anteverted, of normal size, and adhered to the anterior abdominal wall, and the above-said mass was palpable with bilateral forniceal fullness.

Investigations

**Figure 1:**
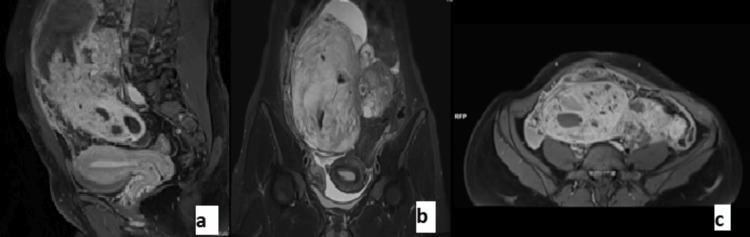
Contrast-enhanced MRI Contrast-enhanced MRI showing multilobulated multicystic lesion of abdomen and pelvis in (a) sagittal view, (b) coronal view, and (c) transverse sectional view.

Outside post-cystectomy, contrast-enhanced computed tomography (CECT) of the abdomen and pelvis done was suggestive of a heterogeneous predominantly solid lesion (16.2 cm x 9.5 cm x 16.3cm) with cystic areas within showing heterogeneous post-contrast enhancement arising from the right adnexa and extending into the abdominal cavity. Among the tumor markers, CA125 was raised to 295 U/ml; carbohydrate antigen 19.9 (CA 19.9), carcinoembryonic antigen (CEA), beta human chorionic gonadotropin (b-hCG), lactate dehydrogenase (LDH), and alpha-fetoprotein (AFP) were normal. Contrast-enhanced magnetic resonance imaging (CE MRI) of the abdomen and pelvis was conducted to confirm the origin, which showed a large well-defined multilobulated multicystic lesion of size 16 cm x 9.3 cm x 16.3 cm arising from the left ovary extending from the pelvis up to the epigastric region with mild ascites, and suspicious deposits measuring 3.8 cm x 1.8 cm were noted in the right pelvic wall as shown in Figure 1. The hemogram, liver, and kidney function tests were normal. A provisional diagnosis of neoplastic adnexal mass was made, and the patient was planned for laparotomy.

Treatment

**Figure 2:**
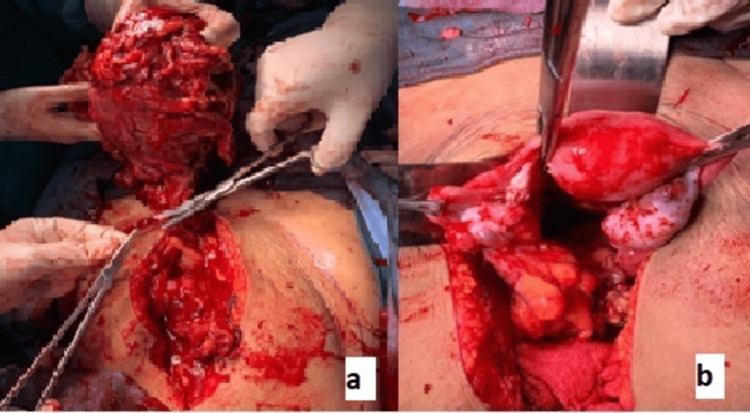
Intraoperative findings (a) A 16 cm x 16 cm necrotic mass with areas of hemorrhage encasing the left mid ureter as seen in laparotomy. (b) Operative finding of a normal uterus, bilateral fallopian tubes, and ovaries.

Upon exploratory laparotomy, a 16 cm x 16 cm necrotic mass was noted in the abdominal cavity, covered by omentum, adhering to the bowel, and encasing the left ureter (Figure 2, Panel a). Another 7 cm x 6 cm hard mass was seen adhered to the left pelvic wall. The uterus, fallopian tubes, and ovaries were healthy with no apparent enlarged lymph nodes (Figure 2, Panel b). Excision of masses was done with a partial left ureteric excision and sent for frozen evaluation, which reported a spindle cell tumor that was probably benign in nature. Assistance from a urologist was sought, and ureteroureterostomy was done after double-J (DJ) stenting. This was followed by omentectomy and bilateral pelvic lymph node dissection.

Histopathology

**Figure 3:**
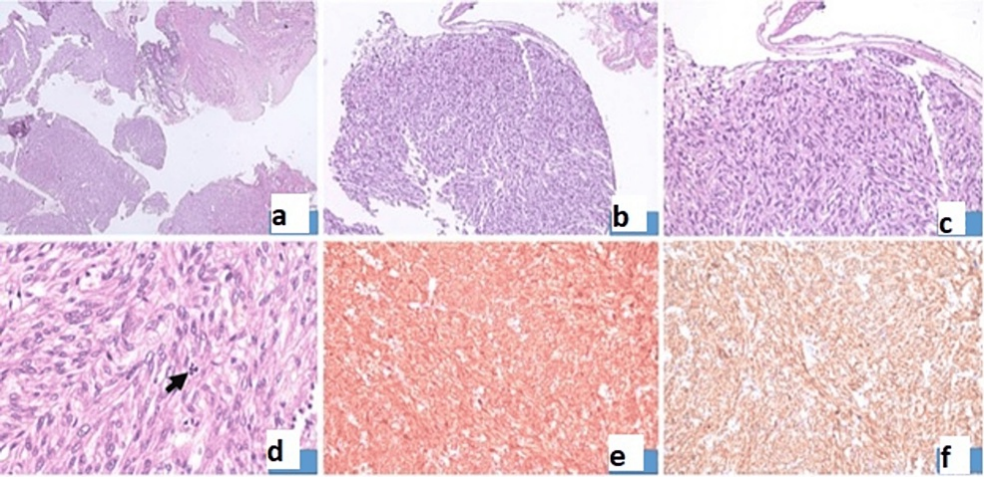
Histopathology images (a) Section showing ureteric lining and a tumor arising and breaching the adventitial layer of the ureter. (b) Section 100x H&E showing a tumor composed of spindle-shaped cells with nuclei being elongated with pointed edges. Nuclear pleomorphism and atypical mitoses are also seen. (c) Section 200x H&E higher magnification of tumor. (d) Section 400x H&E higher magnification of tumor with atypical mitosis seen. (e) Smooth muscle actin (200x) showing strong cytoplasmic staining. (f) Caldesmon (200x) showing strong cytoplasmic staining. H&E: Hematoxylin and eosin.

A gross examination of the larger mass showed multiple friable solid cystic areas and no normal ovarian parenchyma, and the smaller mass revealed grey-white whorled areas with necrosis and myxoid degeneration. Multiple sections studied showed tumor cells arranged in tightly packed fascicles with a storiform pattern with eccentrically placed nuclei and abundant cytoplasm at places. Few areas of necrosis (ischemic tumor), infraction, and hemorrhage were noted. Few foci showed brisk mitosis (10/10 hpf). Thus, the final histopathology was reported as malignant mesenchymal tumor in both masses. The ureter margin showed features of a spindle cell tumor. There was no evidence of malignancy in the omentum and bilateral pelvic lymph nodes. Upon immunohistochemistry, tumor tissue was positive for h-Caldesmon and smooth muscle actin. This suggested FNCLCC (Fédération Nationale des Centres de Lutte Contre le Cancer) grade 2 leiomyosarcoma of smooth muscle origin involving the margin of the ureter. The histopathological findings are shown in Figure 3, Panels a-f.

Outcome and follow-up

Ten days post-surgery, positron emission tomography-computed tomography (PET-CT) showed a dural-based hyperdensity lesion in the right parietal lobe region. This was followed by a CE MRI brain, which revealed a calcified granuloma in the right superior temporal gyrus. Post-op CA 125 was 44.2U/mL.

The case was discussed in the tumor board, and the decision was taken for chemotherapy. She received six cycles of chemotherapy with adriamycin (doxorubicin)-ifosfamide-mesna (AIM) protocol every three weeks sandwiched with 60 Gy radiation in 30 fractions over five weeks with no undue side effects. Upon response assessment, multiple peritoneal deposits were seen on CECT, which was confirmed by biopsy. She has now been planned for second-line chemotherapy.

## Discussion

Leiomyosarcomas are part of a broad group of rare tumors known as soft tissue sarcomas. These soft tissue sarcomas constitute 2.1% of all sarcomas and 1%-2% of all malignant genitourinary tumors [3,4]. Among these, leiomyosarcomas form the largest histological subtype accounting for nearly 50% of cases [5]. Due to the paucity of data, any epidemiological pattern could not be established. In literature, only 20 cases of ureteric leiomyosarcoma have been reported as of now, and in most of these cases, the histopathological diagnosis was confirmed by immunohistochemistry. In all these cases, the age of occurrence was more than 40 years wherein most were females [2,6-8]. The presenting symptom in these studies including ours was pain in the abdomen and flank, while in a study by Shastri [6], it was a lump in the abdomen. Hematuria was also seen to be a chief complaint in a few cases [8].

The cause of this tumor is still not clear due to limited data, although some available data suspect retinoblastoma 1 gene mutation, previous pelvic radiotherapy, and the use of cyclophosphamide to be causative [7]. MRI has the upper hand in the diagnosis of soft tissue sarcoma [9]. Additionally, an intravenous pyelogram is found beneficial in cases where a tumor of renal tract origin is suspected unlike in our case where both CECT and MRI suggested an adnexal mass. The PET scan is of distinct use in recurrent cases to find out the remnant or metastatic tumor.

Leiomyosarcoma due to its high malignancy has a very low five-year survival rate of approximately 55%-60% for all stages of the disease [10]. Tumor grade and size affect the prognosis of soft tissue sarcomas and are included in the American Joint Association Cancer staging system [11]. Histological grading is done by the French Federation of Cancer Centers Sarcoma Group [12]. Being a rare tumor, ureteric leiomyosarcoma is rarely diagnosed preoperatively and is treated by wide excision of the tumor. Leiomyosarcomas need to be differentiated from rhabdomyosarcoma and other spindle cell tumors on histopathology. On immunochemistry, leiomyosarcoma is positive for desmin and smooth muscle actin, while it is negative for epithelial markers such as cytokeratin and epithelial membrane antigen [13]. After surgery, radiotherapy can be given to patients with large tumors and positive margins [14]. In case of metastasis, palliative chemotherapy is considered a good option with agents like doxorubicin, gemcitabine, docetaxel, and ifosfamide. In a study by Spiess et al., 16% of patients had local recurrences, while 53% of patients had metastasis mostly to lung, liver, brain, and bone [15].

In our case, the team was surprised by the preoperative finding of a mass but normal ovaries against the preoperative imaging suggesting an adnexal mass. The history of the left tubo-ovarian mass in the previous surgery could be what steered us and the radiologists to the most common diagnosis of ovarian neoplastic mass. Nonetheless, we could excise the mass completely with the involved left mid ureter followed by a safe end-to-end anastomosis of the ureter with the assistance of urologists. There lies the importance of handling such cases in a facility with multiple specialties.

## Conclusions

Similar to other leiomyosarcomas, ureteric leiomyosarcomas are highly malignant, aggressive, and fast-growing tumors with a poor prognosis. As observed in scarce literature, ureteric leiomyosarcomas occur mostly in women above 40 years. However, this diagnosis should be a differential in a woman of any age group as per the current case. The symptoms vary from just pain in the abdomen to more specific ones including flank pain, hematuria, and voiding complaints. Diagnosis mostly relies on histopathology and immunohistochemistry; hence, a high degree of suspicion is required preoperatively. MRI and intravenous pyelogram aid in the diagnosis of ureteric leiomyosarcoma but are not very sensitive. Treatment should be surgical excision followed by chemotherapy and/or radiotherapy.

## References

[1] Maruyama E, Azuma H, Yamamoto K, Katsuoka Y (2000). [Retroperitoneal leiomyosarcoma found 5 cm in size: a case report]. Hinyokika Kiyo.

[2] Lv C, Chen N, Zhu X, Zhang X, Zhong Z (2008). Primary leiomyosarcoma of the ureter. Asian J Surg.

[3] Stojadinovic A, Leung DH, Allen P, Lewis JJ, Jaques DP, Brennan MF (2002). Primary adult soft tissue sarcoma: time-dependent influence of prognostic variables. J Clin Oncol.

[4] Russo P, Brady MS, Conlon K, Hajdu SI, Fair WR, Herr HW, Brennan MF (1992). Adult urological sarcoma. J Urol.

[5] Lee G, Lee SY, Seo S, Jeon S, Lee H, Choi H, Jeong BC (2011). Prognostic factors and clinical outcomes of urological soft tissue sarcomas. Korean J Urol.

[6] Shastri RK (2016). Primary leiomyosarcoma of the ureter, a case report. IOSR J Dent Med Sci.

[7] Gerald B, Fabian B, Stephen M (2016). Leiomyosarcoma of the distal ureter: a case report. Malta Med J.

[8] Rushton HG, Sens MA, Garvin AJ, Turner WR Jr (1983). Primary leiomyosarcoma of the ureter: a case report with electron microscopy. J Urol.

[9] Panicek DM, Gatsonis C, Rosenthal DI (1997). CT and MR imaging in the local staging of primary malignant musculoskeletal neoplasms: report of the radiology diagnostic oncology group. Radiology.

[10] Dotan ZA, Tal R, Golijanin D, Snyder ME, Antonescu C, Brennan MF, Russo P (2006). Adult genitourinary sarcoma: the 25-year memorial sloan-kettering experience. J Urol.

[11] Edge SB, Compton CC (2010). The American Joint Committee on Cancer: the 7th edition of the AJCC cancer staging manual and the future of TNM. Ann Surg Oncol.

[12] Coindre JM, Terrier P, Bui NB (1996). Prognostic factors in adult patients with locally controlled soft tissue sarcoma. A study of 546 patients from the French Federation of Cancer Centers Sarcoma Group. J Clin Oncol.

[13] Iwata J, Fletcher CD (2000). Immunohistochemical detection of cytokeratin and epithelial membrane antigen in leiomyosarcoma: a systematic study of 100 cases. Pathol Int.

[14] Salerno KE (2022). Radiation therapy for soft tissue sarcoma: indications, timing, benefits, and consequences. Surg Clin North Am.

[15] Spiess PE, Kassouf W, Steinberg JR (2007). Review of the M.D. Anderson experience in the treatment of bladder sarcoma. Urol Oncol.

